# An efficient cell micronucleus classification network based on multi-layer perception attention mechanism

**DOI:** 10.1038/s41598-025-93158-3

**Published:** 2025-03-07

**Authors:** Weiyi Wei, Linfeng Cao, Jingyu Li, Luheng Chen

**Affiliations:** College of Computer Science and Engineering, Northwest Normal University, Lanzhou, China

**Keywords:** MobileViT, Cell micronucleus, Deep learing, Attention mechanism, Computational biology and bioinformatics, High-throughput screening, Image processing

## Abstract

Cellular micronucleus detection plays an important role in pathological toxicology detection and early cancer diagnosis. To address the challenges of tiny targets, high inter-class similarity, limited sample data and class imbalance in the field of cellular micronucleus image detection, this paper proposes a lightweight network called MobileViT-MN (Micronucleus), which integrates a multilayer perceptual attention mechanism. Considering that limited data and class imbalance may lead to overfitting of the model, we employ data augmentation to mitigate this problem. Additionally, based on domain adaptation, we innovatively introduce transfer learning. Furthermore, a novel Deep Separation-Decentralization module is designed to implement the reconstruction of the network, which employs attention mechanisms and an alternative strategy of deep separable convolution. Numerous ablation experiments are performed to validate the effectiveness of our method. The experimental results show that MobileViT-MN obtains outstanding performance on the augmented cellular micronucleus dataset. Avg_Acc reaches 0.933, F1 scores 0.971, and ROC scores 0.965. Compared with other classical algorithms, MobileViT-MN is more superior in classification performance.

## Introduction

Cell micronucleus, abnormal small structures in the cell nucleus, serves as a crucial indicator for early tumor diagnosis and reflects cellular DNA damage. These structures can form due to various factors including genetic variation, environmental exposure, and drug response. Vral A, Fenech et al.^1^ demonstrated this through their analysis of micronucleus frequency in radiation-exposed human lymphocytes using cytokinesis-block assay, while De Almeida et al.^2^ investigated its relationship with clinical characteristics in hepatocellular carcinoma patients. The significance of micronucleus extends to personalized medicine, as evidenced by Torres et al.^3^, who validated its use as a biomarker for monitoring genotoxic damage in radiation-exposed workers.

The ubiquitous presence of micronuclei in diseased cells has made their automatic detection a focal point in medical image analysis. Early approaches relied on manual feature extraction, with notable contributions from several researchers. Toossi^4^ developed an automated scoring system for peripheral blood smear analysis, while Galecki^5^ evaluated scoring accuracy for radiation-induced micronuclei. Lepage^6^ addressed optimization in quantitative microscopy for micronucleus counting, and Sarasa Yano^7^ advanced the field through three-dimensional analysis of brain neuron micronuclei. Zhang^8^ improved cell segmentation accuracy through morphological operations. However, these traditional methods often struggle with the complex structures and noise inherent in medical images.

In recent years, deep learning has been widely studied beacuse of its exceptional learning capability and efficient handling of large-scale data. Some of the well-known deep learning algorithms include AlexNet^9^, GoogLeNet^10^, ResNet^11^, VGG-16^12^, MobileNet^13^, and MAS-Net^14^, etc. These deep learning algorithms leverage large-scale datasets to enable end-to-end training, automatically learning high-level feature representations from input images.However, image analysis of cellular micronuclei presents several unique challenges. First of all, micronuclei are abnormally small structures of the nucleus, and their target size is relatively small relative to the cell as a whole, which makes accurate detection difficult. Secondly, micronuclei and nuclei are highly similar in morphology and grayscale characteristics, which increases the difficulty of classification. In addition, since it takes a lot of time for professionals to acquire annotated micronucleus cell image data, there is less training sample data available. Finally, in practice, the number of micronucleus-containing cells is often much smaller than that of normal cells, resulting in a serious class imbalance.

Existing deep learning methods have made some progress in micronucleus detection. Rodrigues et al.^15^ improved efficiency by using imaging flow cytometry and Amnis^®^ AI software for automated detection of micronuclei. Wills et al.^16^ demonstrated that imaging flow cytometry and deep learning image classification can automate micronucleus scoring. Wei et al.^17^ improved the computer-aided diagnostic method for micronucleus recognition by using AlexNet and visual attention mechanisms. However, these methods mainly focus on the improvement of the overall detection accuracy, and fail to solve the problem of small target detection. Alafif et al.^18^ achieved a classification accuracy of 80.06% using a deep transfer learning approach, but there were still challenges in dealing with samples with high similarity between classes. The deep learning workflow developed by Anand Panchbhai et al.^19^ achieved more than 90% accuracy in micronucleus detection, but did not consider the effect of sample imbalance. The rapid automation software developed by Shen et al.^20^ performs well in handling simple scenarios, but it still needs to be improved for small target detection in complex backgrounds. While the methods proposed by Sabeena et al.^21^ and Lin et al.^22^ have made progress in cell classification, they require large amounts of training data to achieve desired results. Xie et al.^23^ reduced the model complexity by combining ResNet and enhanced MobileNet to construct a GANs network, but the generalization ability in the case of few samples was limited. Although the improved U-Net model based on the attention mechanism proposed by Ma et al.^24^ enhances the learning of non-salient features in the nucleus, it fails to solve the problem of class imbalance. Therefore, aiming at the problems of small target, high similarity between classes, few sample data and class imbalance in cell micronucleus images, this paper proposes a MobileViT-MN lightweight model based on multilayer perception attention mechanism.The main contributions of this paper can be summarized as follows:

1. We propose a novel DD module that effectively addresses the challenge of small target detection and feature preservation in micronucleus classification. This module significantly reduces computational complexity while maintaining high detection accuracy, achieving a remarkable balance between efficiency and performance.
2. We design an innovative dual-attention mechanism that combines the advantages of SE and NAM attention at different levels of the network architecture. This hierarchical attention design enables more precise feature extraction and better handling of morphological variations in micronuclei, leading to improved classification accuracy.
3. We develop an effective transfer learning strategy tailored for medical image analysis, particularly addressing the challenges of limited data availability in micronucleus detection. This approach significantly enhances model generalization and robustness, achieving state-of-the-art performance (Avg_Acc of 0.933, F1 score of 0.971) while maintaining low computational overhead.

## Methods

### Network architecture overview

Vision Transformer (ViT) has shown great potential in computer vision tasks. However, when applying ViT to cell micronucleus detection, several challenges emerge: (1) the large model parameters and high computational requirements make it impractical for deployment in resource-constrained medical environments; (2) the lack of inductive bias in pure transformer architectures leads to insufficient local feature extraction, which is crucial for detecting small micronuclei; (3) the model requires large amounts of training data, which is particularly challenging in medical imaging scenarios.

**Fig. 1:**
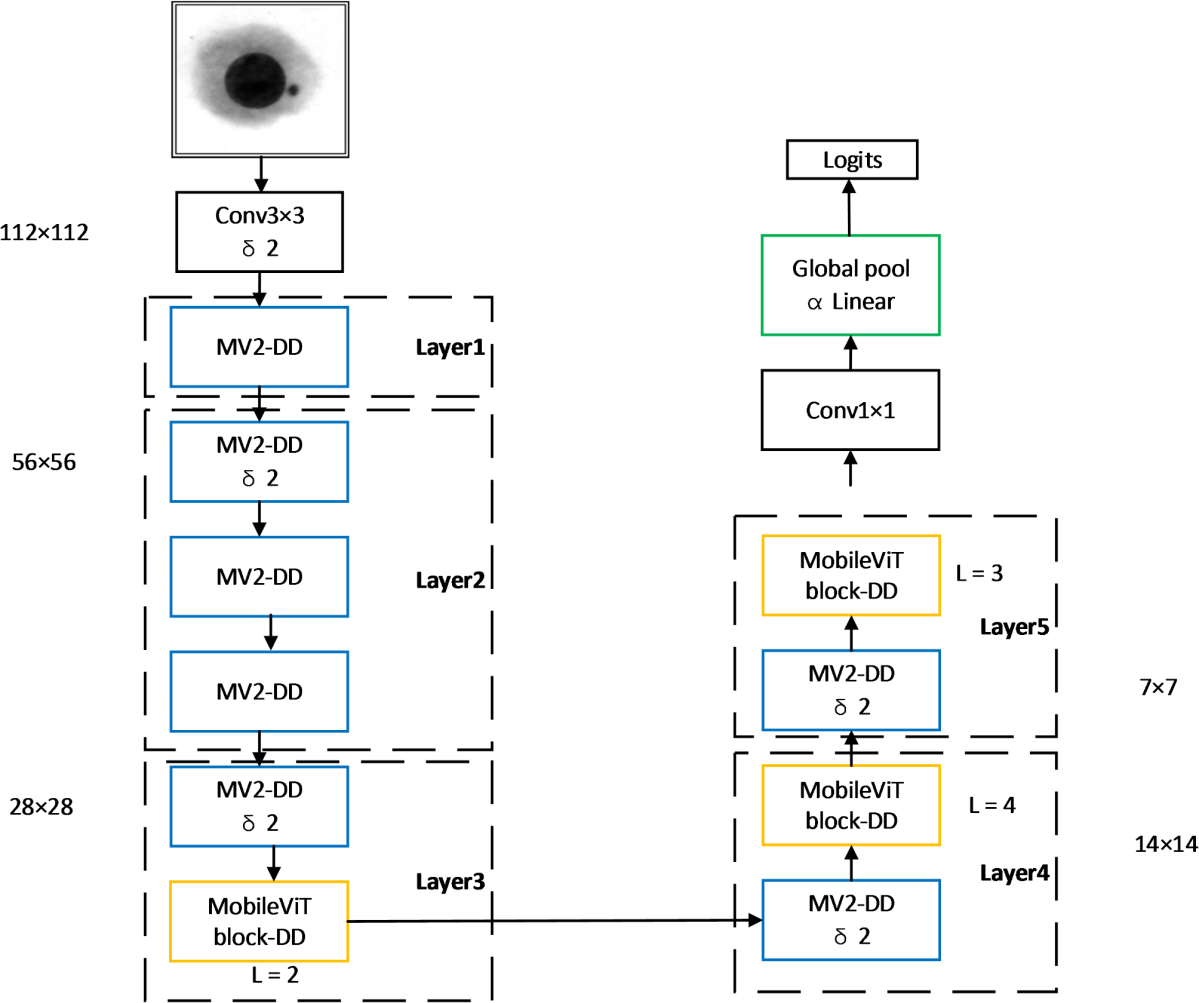
MobileViT—MN.

To address these challenges, we propose MobileViT-MN based on the MobileViT^25^ architecture. Our model incorporates several key innovations: a lightweight CNN-Transformer hybrid architecture to balance computational efficiency and feature extraction capability; specialized attention mechanisms to enhance focus on small micronuclei regions; transfer learning strategies to overcome limited data availability.MobileViT-MN primarily consists of ordinary convolutions, the MV2-DD (MobileNetV2-Deep Separation Decentralization) module, the MobileViT block-DD module, global pooling, and fully connected layers. The MobileViT-MN framework is shown in Fig. 1. MobileViT-MN employs several lightweight convolutional neural networks, namely MV2-DD, as feature extractors to capture low-level and mid-level features from input images. Following the feature extraction using MV2-DD, the image is divided into fixed-size blocks known as ‘patches’. Each patch is flattened into a vector and undergoes a linear transformation to obtain its corresponding embedding representation.

These embedding vectors are then passed as input to the Transformer module. Subsequently, Transformer module captures global contextual information and establishes correlations between images. Each Transformer module consists of multiple encoder layers, with each layer comprising of multi-head self-attention mechanisms and feed-forward neural networks. This design enables the model to capture global information while preserving local relationships within the image. Finally, MobileViT-DD aggregates the features at each location by performing global average pooling to obtain a fixed-length representation of the entire image. Then this representation is input as the fully connected layer of the classifier, which maps image features to specific classes.

### MV2-DD

The detection of cell micronuclei faces challenges in feature extraction in terms of information loss during feature downsampling, which is particularly critical for small micronucleus detection and difficulty in distinguishing micronuclei from similar cellular structures. To address these issues, we propose MV2-DD, which introduces a deep separation-dispersion module based on the SE attention mechanism.This module addressed the information loss issue associated with $\:3\times\:3$ depthwise separable convolutions when capturing finer-grained features, and enabling the model to better focus on more crucial features for the current task. SE^26^ is an attention mechanism used to enhance the performance of convolutional neural networks (CNNs), which aims to automatically learn the relevance weights for each channel in the input feature map. It consists of two steps: squeeze and excitation. In squeeze stage, the global average pooling operation is used to compress the feature map of each channel into a single value to obtain global information. Calculation formula of squeeze operation is as follows :

1 $$Z_{c} = F_{{sq}} (X) = \frac{1}{{H \times W}}\sum\limits_{{i = 1}}^{H} {\sum\limits_{{j = 1}}^{W} {X(i,j)} }$$

where $\:{F}_{sq}$ represents squeeze function, X denotes the input feature map, H and W denote the height and width of the feature map, $\:i$ and $\:j$ denote the coordinate information on the feature map.

In the excitation stage, a small feed-forward neural network (MLP) is utilized to learn a nonlinear transformation function. This function is employed to compute the excitation weights for each channel, allowing for the weighted adjustment of their importance. The formula for the excitation operation is as follows:

2 $${\text{S}} = {\text{F}}_{{excite}} (Z_{c} ) = \sigma (W_{2} \delta (W_{1} Z_{c} ))$$

where $\:{Z}_{c}$ represents the compressed features, $\:{F}_{excite}$ denotes the excitation operation function, σ represents the sigmoid function, δ represents the activation function, W_1_ and W_2_ denote the parameters of the excitation operation.

**Fig. 2:**
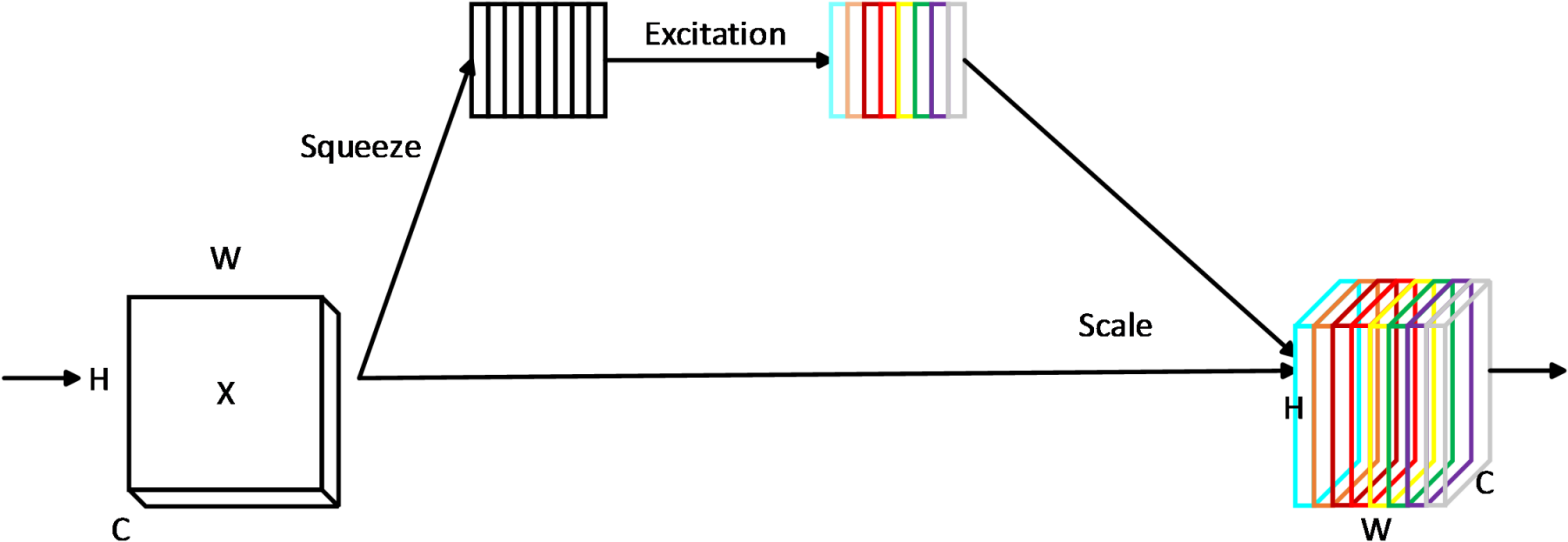
SE attention mechanism.

The deep separation-decentralization module based on the SE attention mechanism possesses several advantages, including adaptability, strong expressive power, and a low parameter count. The adaptability is demonstrated through its ability to automatically learn the weights for each channel based on the characteristics of the input data, thereby enhancing the model’s generalization capability. In terms of expressive power, this module can enhance channels containing important information while suppressing irrelevant ones by adjusting their weights. This aids in capturing the crucial features of the input data more effectively. Furthermore, the SE mechanism requires relatively fewer parameters, as it only needs to learn a small feed-forward neural network to compute the excitation weights for the channels, without the need to learn multiple matrices and vectors. SE attention mechanism shown as Fig. 2.

In our MV2-DD, the input is initially passed through a $\:1\times\:1$ convolution to expand the number of channels, mapping the low-dimensional spatial information into a higher-dimensional space. Then the feature information of the image is extracted by a SEDD (SE-based deep separation decentralization) module, which consists of a $\:3\times\:3$

**Fig. 3:**
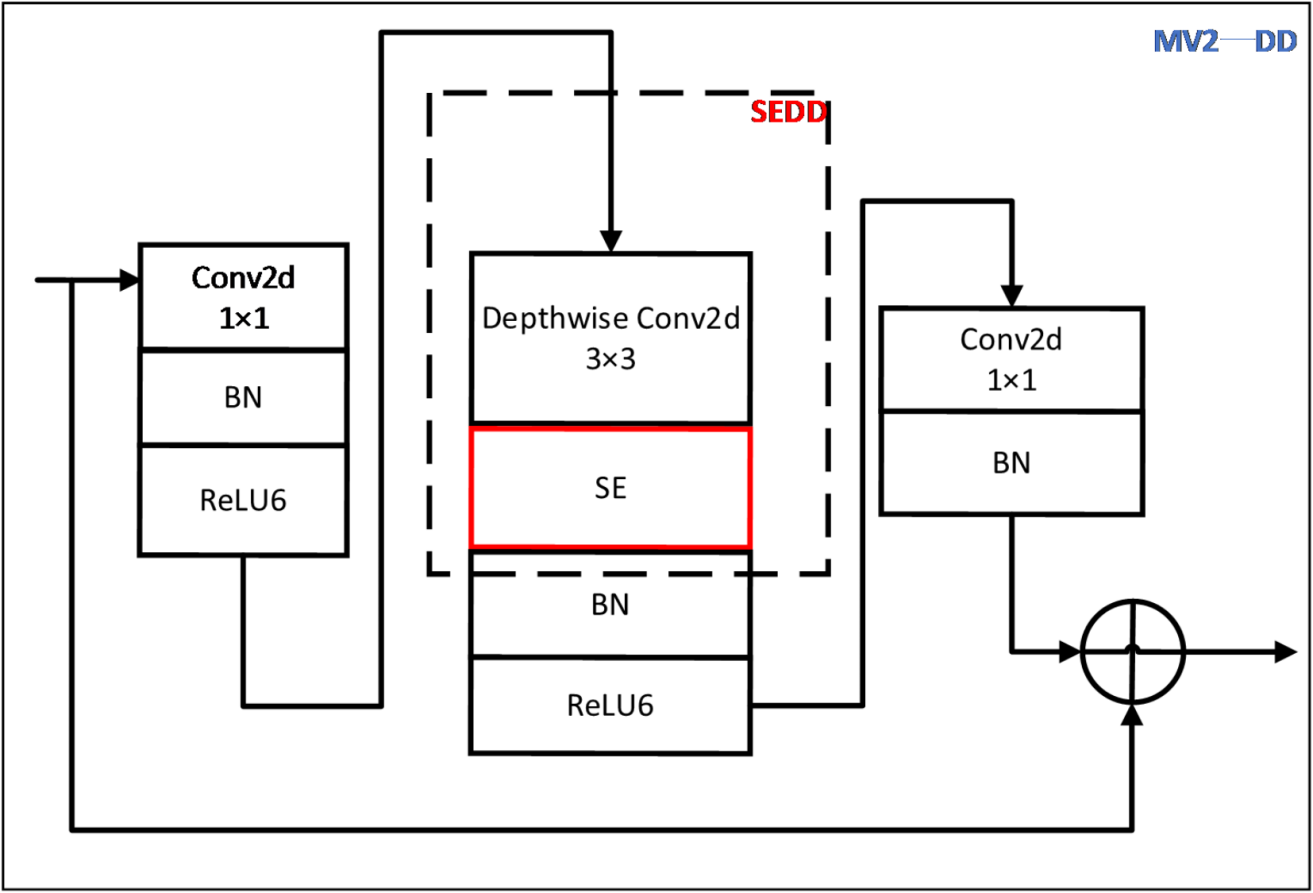
MV2-DD.

depth-wise separable convolution and an SE attention mechanism. Finally, a $\:1\times\:1$ convolution is applied to reduce the number of channels, mapping the obtained high-dimensional features back to the low-dimensional space. MV2-DD framework shown as Fig. 3.

### MobileViT block-DD

Despite its innovative design, applying MobileViT directly to micronucleus detection reveals several critical limitations. Micronuclei exhibit significant size variations across different samples, but MobileViT’s single-scale feature processing struggles to effectively capture this diversity. Additionally, analyzing high-resolution medical images with MobileViT incurs substantial computational costs, limiting its practical deployment in clinical settings. Moreover, the subtle morphological differences between micronuclei and normal nuclear fragments demand meticulous attention to local structural details, which the original architecture fails to adequately address.

To tackle these challenges, our MobileViT block-DD introduces strategic enhancements through an integrated approach. We combine deep amplification with parameter reduction to achieve comprehensive feature extraction while lowering computational demands. The incorporation of NAM-based attention mechanism enables precise focus on distinctive morphological characteristics of micronuclei, while our optimized feature fusion strategy effectively combines both local details and global contextual information. These improvements collectively enable robust detection across varying micronucleus sizes and morphologies while maintaining computational efficiency.

### Deep amplification and parameter reduction

Inspired by Depthwise Separable Convolution (DSC)^27^, we decompose the standard convolution in both local feature extraction and feature fusion parts into two components: a Depth-Conv layer and a PFCL (pixel full connection layer). Depth-Conv performs channel-wise independent convolutions with 1 × 1 kernels, preserving spatial information while maintaining channel independence. PFCL then enables cross-channel information fusion through convolution operations, generating output feature maps with adjustable channel dimensions.

**Fig. 4:**
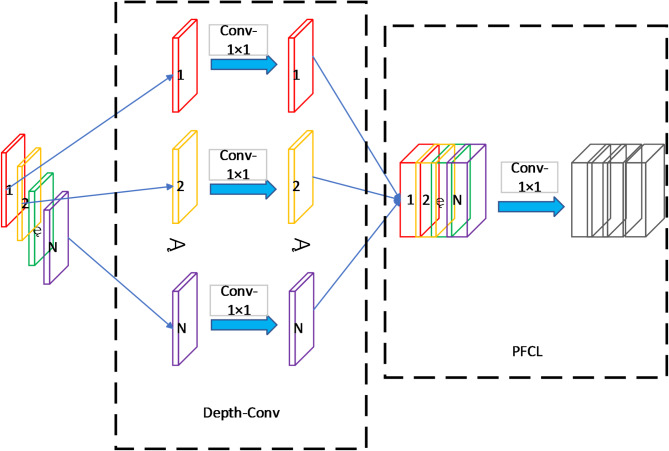
Example diagram of convolutional decomposition.

This improved architecture enhances model expressiveness while significantly reducing computational complexity. Compared to MobileViT’s standard convolutions requiring C kernels for C input channels, our approach needs only C small-sized kernels, resulting in fewer parameters and lower storage requirements. This optimization makes our model particularly suitable for real-time inference on mobile devices and resource-constrained environments, as illustrated in Fig. 4.

### NAMDD

The Normalization-based Attention Module (NAM)^28^ enhances neural networks’ expressive power through feature normalization and selective attention. Its core mechanism normalizes input features and combines them with original features to extract statistical information, which generates attention weights for feature enhancement. In our MobileViT block-DD, we integrate NAM into a deep separation decentralization module to enable selective attention allocation, allowing the model to focus on crucial features while suppressing less relevant information. The following is the corresponding calculation formula, where ${{\hat{X}}}$ represents the normalized features, A represents the calculated attention weights, σ represents the sigmoid function, and $\:{f}_{att}$ represents the convolution operation.

3 $${\text{A}} = \sigma (f_{{att}} (\hat{X}))$$

Furthermore, the normalized features are element-by-element fused with the original features to obtain a feature representation weighted by attention. This process can enhance or suppress different parts of the original features to highlight important characteristics. The following are the corresponding computational formulas, where X represents the input original features, Y represents the attention-weighted features, and $\odot$ represents element-wise fusion.

4 $${\text{Y}} = X \odot \text{A}$$

Finally, the attention-weighted features are re-mapped back to the original feature space through further processing. This step helps the network better utilize the information weighted by attention. The corresponding formula is as follows, where $\:{f}_{recon}$ is a convolution operation used for feature reconstruction. Z represents the final output.

5 $${\text{Z}} = f_{{recon}} (Y)$$

**Fig. 5:**
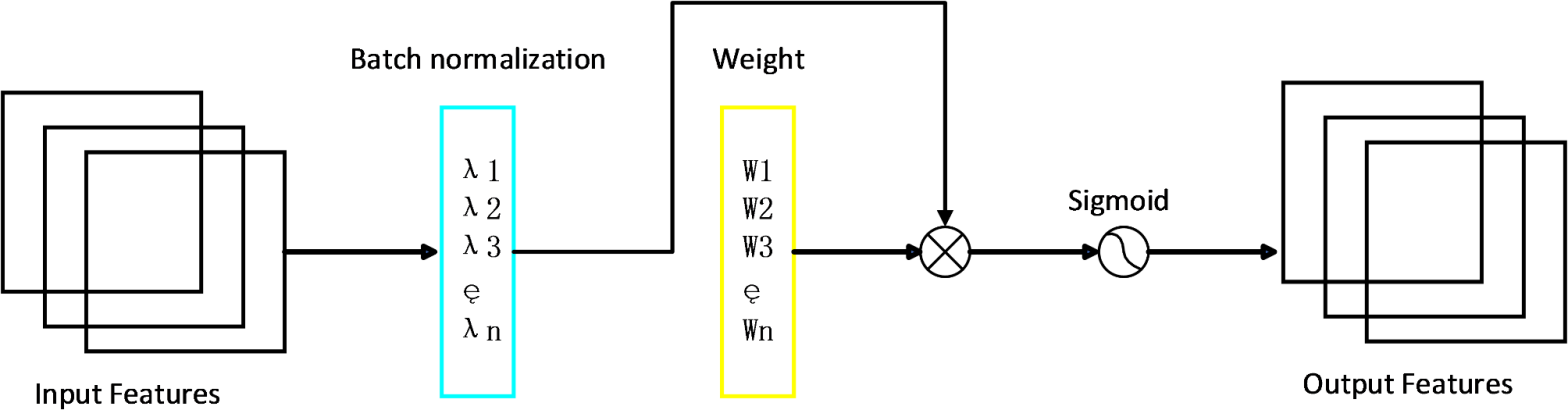
NAM attention mechanism.

The advantage of the NAM-based deep separation decentralization module lies in its utilization of a simple normalization operation to achieve feature weighting, avoiding complex computation processes. This module has fewer parameters and computational requirements, making it suitable for lightweight model design and real-time inference tasks. The NAM attention mechanism shown as Fig. 5.

**Fig. 6:**
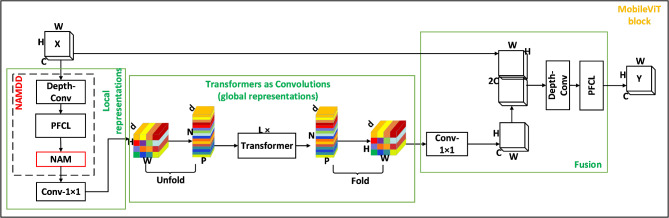
MobileViT block-DD.

In MobileViT block-DD, this paper employs NAM to the local feature construction stage, allowing the model to focus more on important regions in the image, and reduce interference from redundant information, which enhances the importance of the information input to the transformer module and reduces the computational requirements within the complex transformer module. Local Representation processes the input feature map by NAMDD and 1 × 1 convolution to obtain a local feature representation. Global Representation divides the feature map into multiple patches and converts them into sequence form by performing Unfold operation on the local feature representation, and then patches through the transformer to model the local features globally, Afterward, the global feature representation was got by performing Fold operation. Finally, the global feature is fused with the original input features using 1 × 1 convolution, Depth-Conv, and PFCL, resulting in the output of the MobileViT block-DD. The MobileViT block-DD framework shown as Fig. 6.

### Transfer learning and parameter optimization

Transfer Learning applies pre-trained parameters and feature representations to related or unrelated domains, to accelerate model learning and improve performance^29^. Transfer Learning has the advantages of using source domain data to improve model performance, strengthening model generalization ability, and sharing common features to reduce repeated learning in the case of small amount of data or limited resources. MobileViT is a model pretrained on the ImageNet dataset, while ImageNet as an ideal source domain for knowledge transfer.

Due to the differences in input data, output requirements, and domain-specific features among tasks may render the initial model parameters unsuitable for the current task. Therefore, this paper uses training data specific to the task to adjust and optimize parameters, enabling it to better adapt the input and output requirements of the task. Parameter optimization is crucial in machine learning and deep learning as it enables automatic adjustment of model parameters through data-driven learning, thereby enhances the model’s suitability for specific task demands and data distributions.

## Results

### Experimental setup

The TVZD dataset was used in the experiments, where all input images had dimensions of 224 × 224. A batch size of 64 was used for training. Due to the increasing complexity of the network, an initial learning rate of 0.001 was set to ensure rapid convergence during the early stages of training and better convergence to the optimal solution in the later stages. The network was deployed using the PyTorch deep learning framework and pretrained on ImageNet. The training process involved 50 epochs. All the code was implemented in Python3.8 and executed on an RTX 2080 Ti (11GB) GPU.

### Dataset description and partitioning

**Fig. 7:**
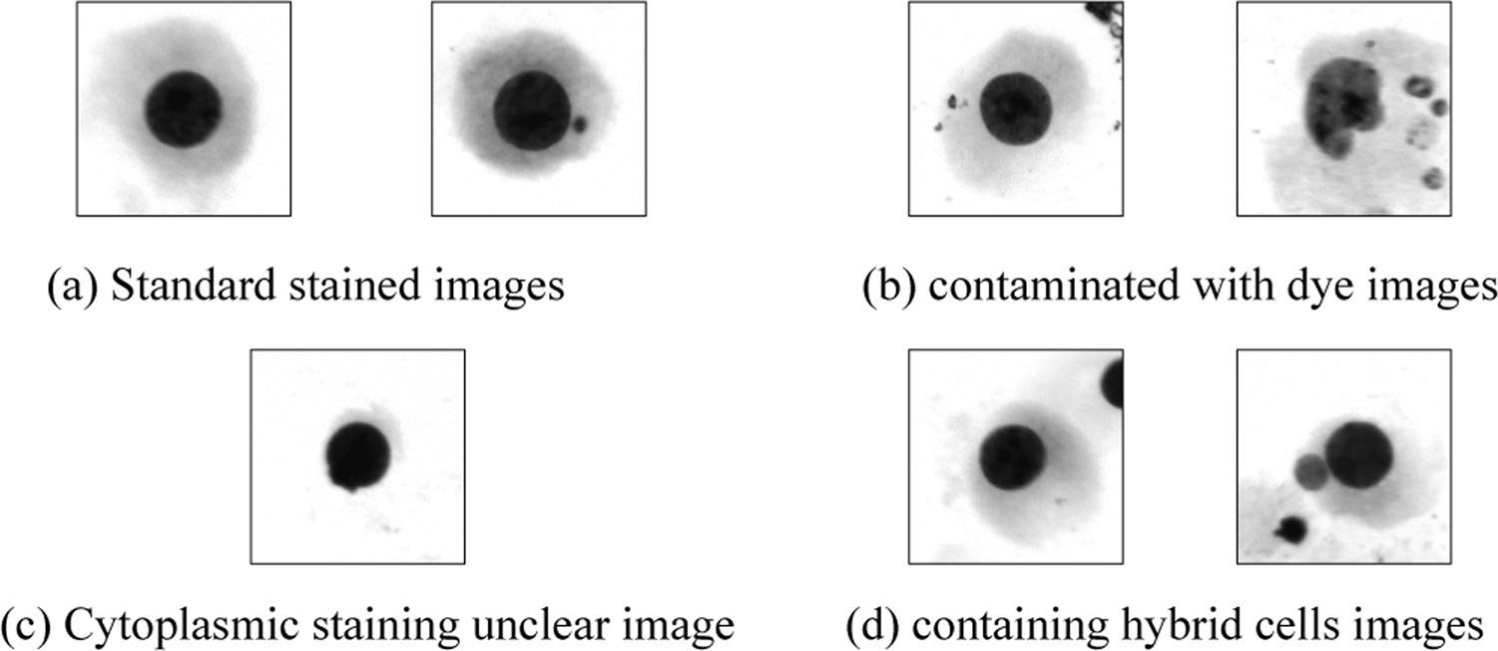
Part data images.

The dataset used in this paper was obtained from the Radiology Laboratory of a provincial Center for Disease Control and Prevention. All images were captured using the Zeiss MetaSystems Metafer slide scanning platform and were pre-annotated by expert physicians. Figure 7 displays part of images from the dataset, which consists of two categories: images with micronucleus in cells and images of normal cells. In the Fig. 7a,b, and d on the left side represent images of normal cells, while images with micronucleus are shown on the right side.

Given the inherent challenges in obtaining medical image datasets, we employ data augmentation techniques including random rotation, flipping, and scaling^30^. The dataset is first split into training, validation, and test sets (6:2:2 ratio). To address class imbalance, we apply augmentation to the minority class (micronucleus images) in the training set while downsampling the majority class. The test set remains unaugmented to reflect real-world distributions. For validation consistency, both classes undergo augmentation in the validation set. This augmented dataset, named TVZD, has its distribution detailed in Table 1.

**Table 1:** Distribution of TVZD.

Dataset	Normal cells	Micronucleus cells	Total
Train set	2191	2180	4371
Val set	10,420	725	11,145
Test set	2083	145	2228

### The influence verification of the attention module

To systematically evaluate the impact of attention mechanisms and their placement within the model architecture, we conducted ten comprehensive experiments. The experimental results, shown in Table 2, investigate both the effectiveness of different attention mechanism combinations and the optimal number of transformer blocks in the MobileViT block-DD module. This investigation aims to achieve an optimal balance between feature extraction capability and computational efficiency for practical deployment on mobile and embedded systems.

Models are denoted using the following conventions: Model_DSC serves as the baseline with decomposition convolution; MV2_SE and MV2_NAM indicate SE and NAM attention mechanisms applied solely in the MV2-DD module, respectively. The suffixes ‘Mb_tr1’ and ‘Mb_tr3’ denote variations in transformer block numbers (1 or 3) within the MobileViT block-DD module. Mb_SE and Mb_NAM represent models with attention mechanisms applied only in the MobileViT block-DD module. Finally, MV2_SE_Mb_NAM and MV2_NAM_Mb_SE indicate dual-attention models with different combinations in the MV2-DD and MobileViT block-DD modules.

**Table 2:** Results of experiments with different attention combinations.

Model	Paramaters	FLOPs	Time	Avg_Acc_Test	ROC	F1
MobileViT	1.272024	2,516,672	0.0694	0.8841	0.9414	0.9358
Model_DSC	**0.970008**	1,912,640	**0.0633**	0.8896	0.9480	0.9390
MV2_SE	1.077880	2,128,384	0.0864	0.9188	0.9586	0.9547
MV2_SE_Mb_tr1	1.077880	2,128,384	0.0799	0.8720	0.8961	0.8634
MV2_SE_Mb_tr3	1.077880	2,128,384	0.0901	0.5039	0.8071	0.3650
MV2_NAM	0.973832	1,912,640	0.0702	0.9128	0.9360	0.9317
MV2_NAM_Mb_tr1	0.973832	1,912,640	0.0691	0.6685	0.7774	0.7137
MV2_NAM_Mb_tr3	0.973832	1,912,640	0.0714	0.9297	0.9650	0.9510
Mb_SE	1.377080	**2,726,784**	0.0742	0.8888	0.9493	0.9041
Mb_NAM	1.272408	2,516,672	0.0684	0.5132	0.9034	0.8197
MV2_SE_Mb_NAM	1.373880	2,719,616	0.1092	**0.9332**	**0.9716**	**0.9650**
MV2_NAM_Mb_SE	1.377336	**2,726,784**	0.0945	0.9023	0.9480	0.9357

Bold is the optimal indicator.

### Ablation experiment

To systematically evaluate each component’s contribution, we conducted comprehensive ablation experiments. The baseline model (Model_no_trans) was trained solely on our dataset, while subsequent variants incrementally incorporated our improvements: transfer learning (Model_trans), depth-separable convolution (Model_trans_DSC), SE attention mechanism in MV2-DD (Model_trans_DSC_SE), and NAM in MobileViT block-DD (Model_trans_DSC_SE_NAM).

As shown in Table 3, each enhancement module contributes positively to the model’s performance, with average accuracy improving progressively from Model_no_trans to Model_trans_DSC_SE_NAM. This systematic improvement demonstrates the effectiveness of our proposed components in enhancing feature representation and learning capabilities, establishing a strong foundation for micronucleus classification tasks.

**Table 3:** Result of ablation experiment.

Model	Avg_Acc_Test	ROC	F1
Model_no_trans	0.5	0.7551	0.6743
Model_trans	0.8841	0.9414	0.9383
Model_no_trans_DSC	0.6254	0.7826	0.7137
Model_trans_DSC	0.8896	0.9480	0.9390
Model_trans_DSC_SE	0.9188	0.9586	0.9547
Model_trans_DSC_NAM	0.9015	0.9434	0.9512
Model_trans_DSC_SE_NAM	0.9332	0.9716	0.9650

### Contrast experiment

This study conducted a comparative evaluation of several convolutional neural network models on cell micronucleus classification task, providing strong reference basis for research in related fields. A comprehensive analysis was performed from various perspectives, including accuracy, F1, ROC, and others. The experimental results are shown in Table 4.

Our method demonstrates excellent performance with an Avg_Acc of 0.933, ROC of 0.965, and F1 score of 0.971. These results can be analyzed from the perspective of architectural design:

**Table 4:** Comparative experimental results.

CNN model	Paramaters	Avg_Acc	F1	ROC
MobileNet^15^	4.24	0.556	0.469	0.842
VGG-16^14^	138	0.845	0.729	0.951
VGG-Att^19^	138.8	0.916	0.715	0.942
GoogLeNet^12^	6	0.832	0.724	0.947
GoogLeNet-Att^19^	6.8	0.819	0.739	0.944
ResNet^13^	23.5	0.884	0.731	0.939
ResNet-Att^19^	24.3	0.892	0.826	0.933
AlexNet^11^	60	0.802	0.711	0.926
Alex-light^19^	59.6	0.847	0.775	0.941
MSA-Net^16^	3.3	0.891	0.796	0.935
Alex-CA^19^	60.7	0.875	0.784	0.942
AALNet^19^	60.2	0.919	0.789	0.951
Our method	1.37	0.933	0.971	0.965

MobileNet’s lower performance stems from its depth-wise separable convolutions, which reduce computational complexity at the cost of compromising spatial feature extraction capability, particularly crucial for small micronucleus detection. VGG-16 (Avg_Acc of 0.845) performs well due to its deep hierarchical structure, but lacks the attention mechanism needed for focusing on small regions of interest. While GoogLeNet and ResNet series achieve good results through inception modules and residual connections, their complex architectures may lead to overfitting on our limited dataset.AlexNet’s mixed performance (Avg_Acc of 0.802, ROC of 0.926, and F1 score of 0.711) indicates effective basic feature extraction but insufficient capability for subtle micronucleus characteristics. AALNet demonstrates the effectiveness of attention mechanisms in cell classification, though not specifically optimized for micronucleus detection.Our MobileViT-MN addresses these limitations through: (1) MobileViT blocks for efficient feature extraction, (2) Deep Separation-Decentralization module targeting small targets, and (3) lightweight design balancing model capacity with limited training data. These architectural choices enable superior performance in handling small targets, high inter-class similarity, and class imbalance challenges specific to micronucleus classification.

### Clinical significance

The performance metrics of our MobileViT-MN model demonstrate significant clinical value in automated micronucleus detection:

Our model’s high ROC value (0.965) indicates reliable discrimination between normal cells and those with micronuclei, crucial for early cancer screening.The high F1 score (0.971) suggests balanced precision and recall, reducing both false positives that could lead to unnecessary medical interventions and false negatives that might miss early-stage abnormalities.The high average accuracy (0.933) enables reliable quantification of micronucleus frequency in radiation-exposed populations.This accuracy level supports precise dose-response assessments in radiation workers and patients undergoing radiotherapy, helping maintain safety standards in occupational and medical radiation exposure.

## Conclusion

In this paper, we propose a lightweight network called MobileViT-MN for accurate classification of cell micronuclei. Based on the MobileViT network that combines the advantages of CNN and ViT, we introduced three key innovations: a novel DD module integrating depth-wise separable convolution, a dual-attention mechanism combining SE and NAM, and an effective transfer learning strategy. These innovations collectively address the challenges of small target detection, feature preservation, and limited data availability in micronucleus classification. Experimental results demonstrate the effectiveness of our approach, achieving superior performance with an Avg_Acc of 0.933, F1 score of 0.971, and ROC score of 0.965, while maintaining a lightweight architecture with only 1.37 M parameters.

Future research will focus on three main directions. First, we plan to conduct comprehensive experiments to quantitatively evaluate the model’s training efficiency and robustness under various conditions. Second, we will investigate advanced techniques for handling imbalanced data in small sample scenarios, aiming to develop more effective strategies for micronucleus classification. Finally, we will explore methods to further optimize the model architecture, seeking a better balance between computational complexity and detection accuracy. These investigations will contribute valuable insights to both medical image analysis and cellular research domains.

## Data Availability

The datasets analysed during the current study are not publicly available due [There is a confidentiality agreement between the hospital that provides the experimental data and us] but are available from the corresponding author on reasonable request.
